# TMS-induced neural noise in sensory cortex interferes with short-term memory storage in prefrontal cortex

**DOI:** 10.3389/fncom.2014.00023

**Published:** 2014-03-05

**Authors:** Tyler D. Bancroft, Jeremy Hogeveen, William E. Hockley, Philip Servos

**Affiliations:** Department of Psychology, Wilfrid Laurier UniversityWaterloo, ON, Canada

**Keywords:** short-term memory, working memory, scalar memory, TMS, vibrotactile, noise, computational modeling

## Abstract

In a previous study, Harris et al. (2002) found disruption of vibrotactile short-term memory after applying single-pulse transcranial magnetic stimulation (TMS) to primary somatosensory cortex (SI) early in the maintenance period, and suggested that this demonstrated a role for SI in vibrotactile memory storage. While such a role is compatible with recent suggestions that sensory cortex is the storage substrate for working memory, it stands in contrast to a relatively large body of evidence from human EEG and single-cell recording in primates that instead points to prefrontal cortex as the storage substrate for vibrotactile memory. In the present study, we use computational methods to demonstrate how Harris et al.'s results can be reproduced by TMS-induced activity in sensory cortex and subsequent feedforward interference with memory traces stored in prefrontal cortex, thereby reconciling discordant findings in the tactile memory literature.

## Introduction

Vibrotactile short-term memory [often referred to as vibrotactile working memory (VWM)] is a powerful paradigm for studying the behavioral and neural correlates of working and short-term memory (Bancroft et al., 2011a). VWM tasks usually involve presenting subjects with two vibrational stimuli delivered to the hand (the target and the probe), separated by an unfilled delay period, and instruct subjects to report whether the two stimuli are of same or different frequencies, or whether the probe is of a higher or lower frequency than the target. Notably, the salient stimulus feature (vibrational frequency) can be represented as a scalar value, and the firing rates of neurons encoding vibrotactile stimuli tend to be monotonic functions of stimulus frequency (Romo et al., 1999; Romo and Salinas, 2003). This makes vibrotactile memory a useful paradigm for integrating research results across various research methodologies, and recent studies have taken advantage of this property by demonstrating that it is possible to decode the stimulus frequency held in memory from beta-band EEG activity in frontal cortex (Spitzer et al., 2010, 2014; Spitzer and Blankenburg, 2011, 2012). Intriguingly, recent research has suggested that vibrotactile memory may be one of a family of scalar short-term memory tasks, including auditory memory for pure tones and memory for the frequency of visual flicker (Spitzer and Blankenburg, 2012), as well as stimulus amplitude and duration (Spitzer et al., 2014), that appear to share a similar, supramodal neural code in both sensory cortex and higher cortical regions, and rely on the same region of prefrontal cortex as a storage substrate.

An intriguing study, however, poses a challenge to this interpretation of results. Harris et al. (2002) presented subjects with two vibrotactile stimuli, separated by an unfilled delay period, and asked them to compare the stimuli. During the delay period, they applied single-pulse transcranial magnetic stimulation (TMS) to primary somatosensory cortex (SI). This study employed a “virtual lesion” design, in which TMS-induced changes in behavior suggest a causal relationship between peri-stimulation neural activity and task-related perceptual and cognitive functions (Robertson et al., 2003). Harris et al. (2002) found a significant decrease in performance when the TMS pulse was applied to contralateral SI (relative to ipsilateral SI) 300 or 600 ms into a 1500 ms delay period, but not when it was applied 900 or 1200 ms into the delay period. (Note that while the decrease in performance in response to the 900 ms onset TMS pulse did not reach statistical significance (*p* = 0.16), a trend is visible.) In contrast, TMS to ipsilateral SI did not significantly reduce performance. Harris et al. suggested that contralateral SI acts as a memory storage system for VWM. Such a notion is consistent with a previous single-cell recording study that reports SI encoding of complex tactile stimuli (Zhou and Fuster, 1996).

However, this notion conflicts with recent findings from human EEG studies and single-cell recording in non-human primates. Various studies by the research group of Romo et al. have suggested that regions in prefrontal cortex are the storage substrate used during VWM tasks and that no representation of the stored stimulus persists across the delay period in SI (see Romo and Salinas, 2003, for a review), and recent EEG studies by Spitzer and colleagues have reported being able to decode the frequency of a stored vibrational stimulus from prefrontal beta-band activity during the delay period of VWM (and other scalar STM) tasks (Spitzer et al., 2010, 2014; Spitzer and Blankenburg, 2011, 2012). The apparent incompatibility of these findings and those of Harris et al. (2002) raises questions about the scalar memory interpretation of results from VWM research, and also about whether the neural systems underlying VWM differ between humans and non-human primates.

The location of VWM storage has important implications for working and short-term memory theory, and the factors that determine storage location are unresolved. Postle (2006) suggested that stimuli tend to be stored in relevant regions of cortex that have pre-existing representations of that type of stimulus, such as sensory cortex; in order to account for recent experimental findings (including those around vibrotactile memory), we have recently suggested that less complex stimuli with simple neural codes instead tend to be stored in prefrontal cortex (Bancroft et al., 2014). As this theoretical framework is partly based on research showing prefrontal storage of scalar stimuli, reconciling Harris et al.'s (2002) results with other findings (i.e., Romo and Salinas, 2003; Spitzer et al., 2010) has theoretical importance.

We offer an alternative interpretation of Harris et al.'s (2002) findings. According to the former view, the application of TMS suppressed neural activity within SI during the delay period, and the consequent impact on VWM performance can be interpreted as evidence that SI is involved in VWM storage. However, beyond local changes in cortical activity, TMS can induce distal effects at brain regions receiving feedforward inputs from the targeted brain region (e.g., Paus et al., 1997). Rather than SI being a storage medium for vibrotactile memory, we suggest that the application of TMS induces or increases activity in sensory cortex (both in SI and in secondary somatosensory cortex (SII), via feedforward connections), and that this activity then interferes with VWM storage in PFC.

It has been established that TMS can induce neural activity when applied to some areas of sensory cortex, including somatosensory cortex (Sugishita and Takayama, 1993; Ray et al., 1998; Stewart et al., 2001; Ptito et al., 2008). As well, recent behavioral and computational studies have suggested that when irrelevant vibrotactile stimuli are presented during the maintenance period of a VWM task, they reduce performance by being encoded into memory (Bancroft and Servos, 2011; Bancroft et al., 2011b, 2012, 2013). As there is a direct mapping between induced activity in SI and the frequency of the stimulus perception created by that induced activity (e.g., Romo et al., 1998), it follows that increased activity in SI due to TMS could have similar effects to irrelevant somatosensory stimuli.

Perhaps most compellingly, somatosensory memory studies that have used TMS to increase activity in the middle frontal gyrus (a region of prefrontal cortex known to inhibit activity in SI) have reported decreased response times when TMS was applied early (300 ms onset) but not late (1200 ms onset) in the delay period, suggesting a decrease in interference (Hannula et al., 2010; also see Savolainen et al., 2011). Given that these TMS manipulations, known to suppress activity in SI, have been shown to *improve*, not reduce, performance on tactile memory tasks, it raises an interesting question: Is Harris et al.'s (2002) manipulation suppressing activity in SI, or is it producing excitatory or facilitatory effects that impact storage systems further downstream?

In the present study, we adapted a computational model of prefrontal cortex (Miller and Wang, 2006) in order to demonstrate that Harris et al.'s (2002) results can be produced by TMS-induced activity in sensory cortex, resulting in interference with information stored in prefrontal cortex. As pointed out by Miller and Wang, feeding noise into an integrator causes a decrease in performance proportional to the duration of noise. In the present study, the accumulation of noise in PFC leads to an inverse relationship between task performance and the delay between TMS offset and probe onset.

## Model

The model used in the present study was originally developed by Miller and Wang (2006) as a model of prefrontal neurons involved in VWM tasks. We have previously adapted it to model the interfering effects of distractor stimuli on VWM (Bancroft et al., 2013). It is a rate model, based on the interaction of pairs of populations of prefrontal neurons. While the Miller and Wang model operates at a relatively high level of abstraction, it captures the fundamentally subtractive nature of the stimulus comparison process (Romo and Salinas, 2003), and has proven capable of fitting a variety of experimental data (e.g., Bancroft et al., 2013). In addition, the model can be fit to data with relatively few free parameters, which is beneficial when fitting a dataset with relatively few data points (such as the Harris et al. data we consider in this paper).

In this model, comparison (*C*) populations receive input from sensory cortex and have excitatory outputs to populations of memory (*M*) neurons. Memory populations have excitatory self-connections (allowing persistent activity in the absence of external input), and inhibitory connections to *C* populations. The equations governing the behavior of the network are as follows:

(1) $$dr_{C}/dt=\left(\text{1}/\tau \right)(-r_{C}+w_{MC}r_{M}+w_{IC}I)$$

(2) $$dr_{M}/dt=\left(\text{1}/\tau \right)\left(-r_{M}+w_{MM}r_{M}+w_{CM}r_{C}\right)$$

where *r* is the firing rate of a population, τ is a time constant, *w_AB_* represents the strength of a connection from a population *A* to another population *B*, and *I* is the input received from sensory cortex. The addition of *w_IC_* to the model is intended as a potential scaling factor to allow presentation of stimulus frequencies outside of biologically-realistic firing rates (for example, auditory stimuli in the kHz range).

Note that if *w_MM_* is set to 1, the *M* population becomes a perfect (i.e., lossless) integrator, and the equation governing behavior of *M* populations can be reduced to:

(3) $$dr_{M}/dt=\left(\text{1}/\tau \right)\left(w_{CM}r_{C}\right)$$

We have used this reduced equation in the present study.

Upon presentation of a target stimulus, a *C* population transmits the stimulus frequency to an *M* population. The *M* population then inhibits activity in the *C* population, driving the *C* firing rate back to baseline. The self-connection allows the *M* population to maintain its firing rate in the absence of external stimulation. Upon presentation of the probe stimulus to the *C* population, the combination of inhibitory input from the *M* population and excitatory input from sensory cortex results in the *C* population calculating some function of *f_target_* - *f_probe_*, consistent with experimental findings (Romo and Salinas, 2003), and also consistent with decision-making mechanisms used in abstract mathematical models of VWM (Bancroft et al., 2012). Note that experimental findings have reported finding neurons in sensory cortex that have firing rates that are positive monotonic functions of stimulus frequency, as well as neurons that have negative monotonic functions of stimulus frequency (Romo and Salinas, 2003). This plays an important role in the functioning of the model. *C* populations that receive positive monotonic input (we refer to these as *C_+_* populations) will fire above baseline when the probe stimulus is a higher frequency than the target stimulus, while populations that receive negative monotonic input (*C_−_ populations*) will act as detectors for lower-frequency probes.

We have also added decision (*D*) populations to the model to facilitate decision-making. The *D* populations receive excitatory output from *C* populations during the presentation of probe stimuli:

(4) $$dr_{D}/dt=\left(\text{1}/\tau \right)\left(w_{CD}r_{C}\right)$$

During target presentation and the delay period, *w_CD_* is set to 0, and only assumes a non-zero value upon presentation of the probe stimulus. During probe presentation, the *D* populations act as perfect integrators of the activity of the relevant *C* population; this allows a direct comparison between the total activities of the *C_+_* and *C_−_* populations (and therefore the probe-higher and probe-lower detectors).

**Figure 1:**
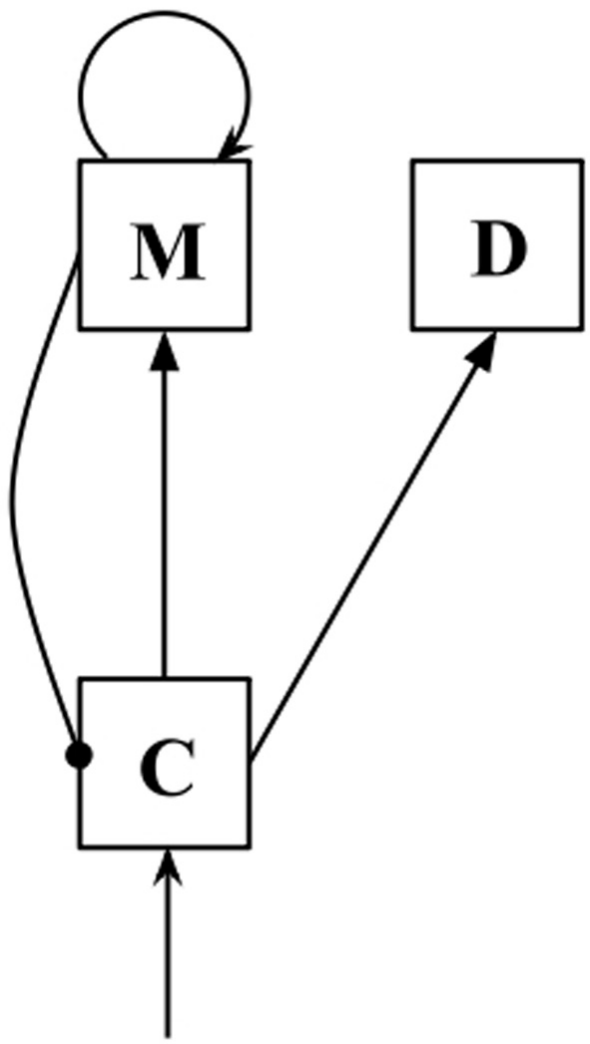
**Diagram of a C/M/D triplet**. Arrows indicate excitatory connections, while lines ending in circles indicate inhibitory connections.

In the present study, we simulated two triplets of *C*/*M*/*D* populations (see Figure 1), one receiving positive monotonic input (with subscript +), the other receiving negative monotonic input (with subscript −). The triplets were not connected to each other. To determine a simulated response, we compared the activity of the *D_+_* and *D_−_* populations shortly after probe offset. If activity in the *D_+_* population exceeded that in the *D_−_* population, it follows that overall activity in the *C_+_* population exceeded that in the *C_−_* population across the probe presentation period, and we recorded a probe-higher response. If activity in the *D_−_* population exceeded that of the *D_+_* population, we recorded a probe-lower response, and if activity in the two populations was equal, a response was randomly chosen.

During the delay period, the model received constant input, with input values drawn from an exponential distribution with the distribution parameter λ, inversely proportional to the mean and variance of the distribution. This noisy input represents ongoing, baseline activity in sensory regions. Critically, we modeled the application of TMS to sensory cortex by allowing λ to vary as a free parameter. If TMS increases activity in sensory cortex, we would expect the magnitude of the noise to increase (and therefore the value of λ to decrease). Further, allowing values of λ to vary separately for ipsilateral and contralateral stimulation allows us to test for differing effects of inhibition depending on laterality—if ipsilateral SI is more greatly inhibited than contralateral SI, we would expect a smaller magnitude of interference (and therefore a greater value for λ). The exponential distribution was chosen for this study as it has one parameter that determines both the mean and the variance of the distribution.

## Simulation methodology

In the present study, input to PFC was of two types. During target and probe presentation, *C_+_* populations received input equal to *w_IC_f*, and *C_−_* populations received input equal to *w_IC_*(40 - *f*), where *f* was the frequency (in Hz) of the stimulus, and *w_IC_* was the strength of the connection from sensory cortex to prefrontal cortex. Consistent with previous work (Bancroft et al., 2013), stimulus frequency (*f*) was drawn from a Gaussian distribution with a mean equal to that of the presented stimulus, and standard deviation (σ) allowed to vary as a free parameter, in order to account for inaccuracy in the neural signal introduced during neural transmission and processing. Firing rates (*r*_population_) were not allowed to decrease below zero. Other parameter values are presented in Table 1.

**Table 1:** **Simulation parameters**.

**Parameter**	**Value**
*f*_target_	20 Hz
*f*_probe, higher_	22 Hz
*f*_probe, lower_	18 Hz
Stimulus duration	1000 ms
Delay period duration	1500 ms
τ	10
*w_IC_*	0.4
*w_CM_*	0.4
*w_MC_*	−0.4
*w_CD_*	0 (during target presentation/delay periods); 0.5 (during probe presentation)
*r*_minimum_	0

During the delay period, *C* populations received noisy input drawn from an exponential distribution at each integration timestep, with the distribution parameter λ set as a free parameter. The parameter λ determines the mean (1/λ) and variance (1/λ^2^) of an exponential distribution.

Harris et al. (2002 Exp. 2) presented subjects with two 1000 ms vibrotactile stimuli (the target and probe), separated by a 1500 ms delay period. TMS was applied to either ipsilateral or contralateral SI, at an onset of either 300, 600, 900, or 1200 ms into the delay period. The target and probe stimuli differed by ±2 Hz, and subjects were required to report whether the probe was of a higher or lower frequency than the target.

To simulate the effects of TMS, λ was allowed to assume two values during the delay period: The initial value (λ_*baseline*_), and a new value upon the application of TMS (λ_*TMS*_). Pilot studies were performed to estimate approximate parameter ranges (based on minimizing error between experimental and simulated results), after which the σ parameter was allowed to vary freely within the range (1.00, 3.00), with a stepsize of 0.5; λ_*baseline*_ was fixed at 0.5, and λ_*TMS* (*ipsilateral*)_ and λ_*TMS* (*contralateral*)_were varied across the range of (0.5, 0.025), taking possible values of 0.5, 0.375, 0.25, 0.125, 0.1, 0.075, 0.05, and 0.025. Two thousand trials were simulated for each combination of onset time and free parameter values. Parameter fit was assessed by minimizing the sum of squared error (*SS*) between the experimental results from Harris et al. (2002) (rounded to four places) and simulated results, and the selected parameter values were those that minimized total *SS* across both ipsilateral and contralateral TMS conditions. (Note that parameter selection was constrained by requiring the value of σ to be the same for both ipsilateral and contralateral stimulation conditions).

To improve the model fit, a second round of simulations was performed based on the best-fitting parameters from the first round of simulations [σ = 2.00, λ_*TMS* (*ipsilateral*)_ = 0.375, and λ_*TMS* (*contralateral*)_ = 0.125]. The value of σ was set to 2.00, and λ_*TMS* (*ipsilateral*)_ and λ_*TMS* (*contralateral*)_varied within the ranges (0.425, 0.325) and (0.175, 0.075), respectively, with a stepsize of 0.025.

Simulations were performed with code written in Python, with the NumPy and standard Python *random* libraries (specifically, *random.expovariate* for the generation of noisy input). Integration was performed using a 4th-order Runge-Kutta, with an integration stepsize of 0.5.

## Results and discussion

**Figure 2:**
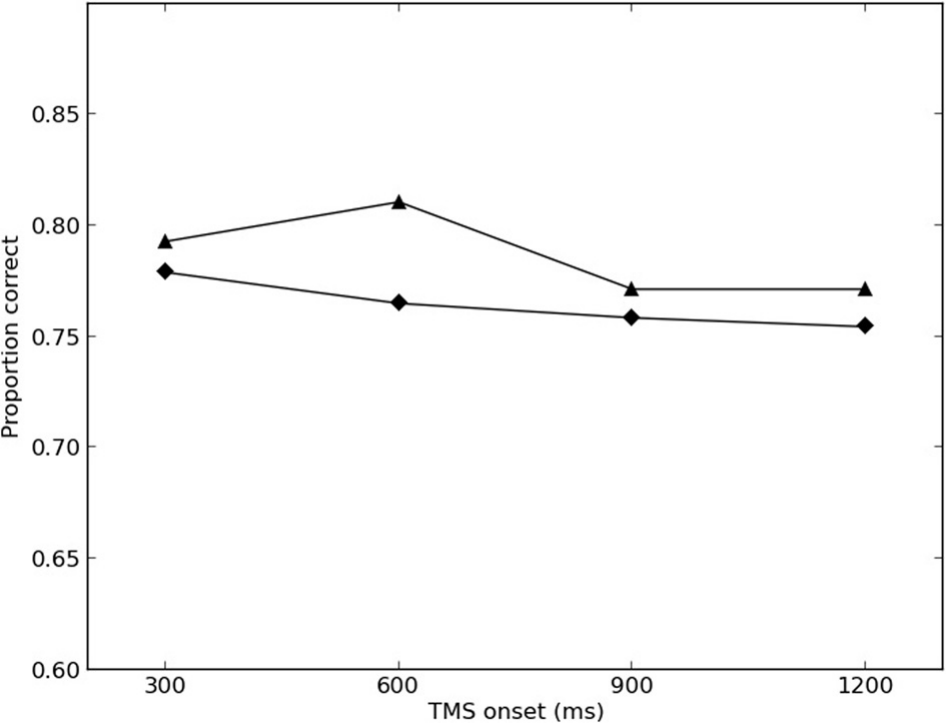
**Simulated and empirical results of TMS to ipsilateral SI**. Triangles denote results from Harris et al. (2002)(Exp. 2); diamonds denote simulated results.

**Figure 3:**
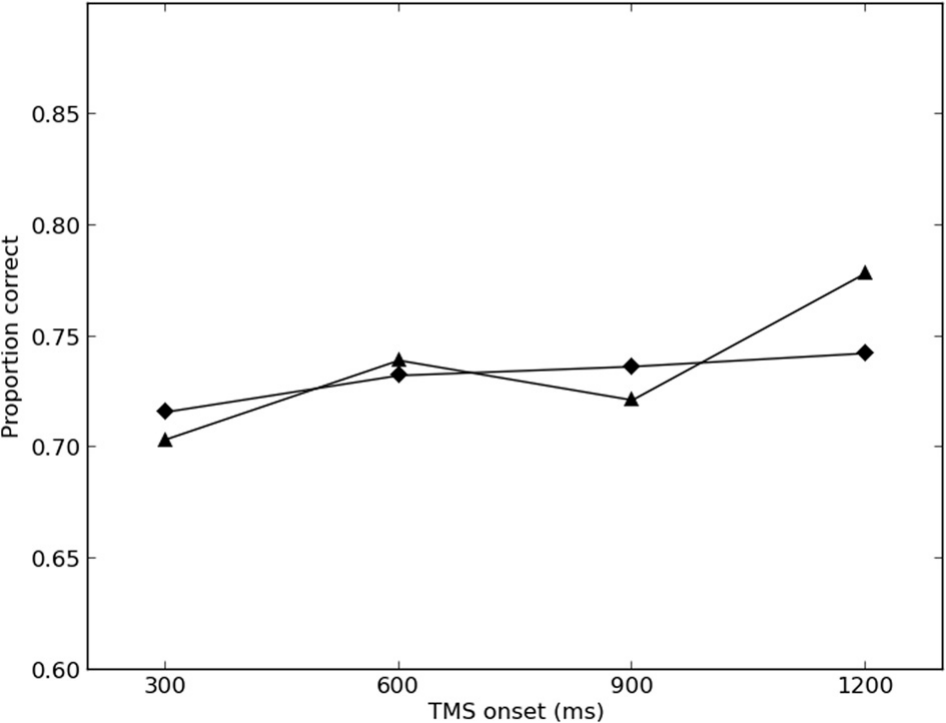
**Simulated and empirical results of TMS to contralateral SI**. Triangles denote results from Harris et al. (2002) (Exp. 2); diamonds denote simulated results.

The results of the final round of simulations are presented in Figures 2, 3. The best-fitting parameter values were found to be σ = 2.0, λ_*TMS* (*ipsilateral*)_ = 0.350, and λ_*TMS* (*contralateral*)_ = 0.150. The *SS* for the best fit was found to be 0.00446 (0.00273 for the ipsilateral condition, and 0.00173 for the contralateral condition), and the variance explained by the model (*r*^2^) was calculated to be 0.780.

Model performance was largely robust against changes in parameter values, with maximum overall *SS* of 0.0962 in the final round of simulations (0.00485 for the ipsilateral condition, and 0.0914 for the contralateral condition).

The results of the present simulation suggest that Harris et al.'s (2002) results can be replicated by assuming that TMS increases activity in sensory cortex, which then interferes with the contents of memory, held in PFC. This interpretation is consistent with the single-cell electrophysiology and EEG literatures (e.g., Romo and Salinas, 2003; Spitzer et al., 2010, 2014; Spitzer and Blankenburg, 2011, 2012), and requires no need to suggest that SI is involved in vibrotactile memory storage.

One crucial part of Harris et al.'s argument was that TMS to SI ipsilateral to the hand receiving vibrotactile stimulation did not produce effects on task performance. They suggested that if VWM storage relied (at least in part) on areas further downstream, such as SII (which possesses bilateral receptive fields), TMS to ipsilateral cortex would produce similar effects to TMS to contralateral cortex. However, recent EEG and MEG studies of tactile memory have reported greater alpha-band activity over ipsilateral SI than over contralateral SI (Haegens et al., 2010, 2012; Spitzer and Blankenburg, 2011). Further, when irrelevant stimuli were expected to be presented to the opposite hand, pre-stimulus alpha power in cortex varied with laterality (Haegens et al., 2012). As alpha-band activity is believed to be linked to inhibitory activity (e.g., Rihs et al., 2007; Haegens et al., 2011), Haegens et al. (2012) suggested that activity in ipsilateral SI could be suppressed in order to inhibit the processing of irrelevant sensory input. In this case, the failure to find effects of ipsilateral TMS does not necessarily reflect a reliance on contralateral SI for VWM storage, but rather may reflect differences in endogenous inhibitory activity between ipsilateral and contralateral sensory cortex.

The results of the present study have an impact reaching beyond the VWM literature. Postle (2006) introduced the emergent-property model of working memory, which suggests that working memory does not rely on a specialized neural system, but rather the interaction between neural systems that primarily serve other sensory, cognitive, or action-related functions. Indeed, Postle explicitly argues that PFC is not involved in the storage of information. For example, task-relevant sensory cortex has been suggested as the storage medium for working memory, and recent neuroimaging studies that have applied novel methods for decoding the contents of sensory cortex have reported finding stimulus information in early visual cortex during the maintenance period of visual memory tasks (e.g., Serences et al., 2009; Christophel et al., 2012). Other, similar results exist.

However, there is an increasing body of evidence that PFC is the storage substrate for simple stimuli and novel stimuli (e.g., Freedman et al., 2001; Bancroft et al., 2014). Perhaps most persuasive are recent studies that have reported decoding the contents of short-term or working memory from prefrontal beta-band activity, regardless of whether the stored aspect of the stimulus was delivered as a tactile vibration, auditory tone, visual flicker (Spitzer et al., 2010; Spitzer and Blankenburg, 2012), or stimulus intensity or duration (Spitzer et al., 2014). While the emergent-property model is compelling, in that it is simple, parsimonious and able to explain a wide variety of results from the literature, when combined with previous findings, the results of the present study suggest that VWM research may require an expansion of the emergent-property model. We have recently suggested that the complexity of a stimulus is at least a partial factor in determining what neural storage systems are recruited (Bancroft et al., 2014).

We acknowledge that the timecourse of the effects of TMS to SI are not well-understood. Indeed, the effects of TMS to SI are not well-understood in general. Harris et al. (2002) selected a target in SI by using TMS to identify the region of greatest tactile extinction, which could be interpreted as evidence of an *inhibitory*, rather than excitatory effect of TMS. However, other research has found excitatory or facilitatory effects of TMS over sensory and motor cortex (Gerwig et al., 2003; Ragert et al., 2004, 2008), and even combined excitatory and inhibitory effects (Oliveri et al., 2000; Moliadze et al., 2003; Strafella et al., 2004). Indeed, even inhibitory effects on neurons in a stimulated region can produce increased neural activity or excitability, due to a reduction in the activity of inhibitory interneurons. Further, Amassian et al. (2002) suggest that single-pulse TMS can excite a large number of neurons in sensory cortex without the effects reaching consciousness.

Effects are also likely to depend heavily on cortical structure and connectivity. Identical stimulation parameters can produce excitation or inhibition in different cortical regions (Paus, 2005), and there is growing evidence that the effects of TMS over a cortical region are state-dependent, with effects possibly depending on pre-existing activity in the region (Harris et al., 2008; Pasley et al., 2009; Abrahamyan et al., 2011). Recently, a number of authors (including Harris) have suggested that TMS resulting in what appears to be inhibitory behavioral effects can actually be due to increased neural excitability resulting in an unfavorable signal-to-noise ratio (Silvanto and Muggleton, 2008; Miniussi et al., 2013).

Whether TMS-induced behavioral results are driven by cortical inhibition, an unfavorable neuronal signal-to-noise ratio, or both to some extent, the present work highlights another critical issue: the local vs. remote interpretation of the neural intervention. Combined TMS/fMRI studies have shown that, even at relatively low intensities, TMS modulates hemodynamic activity in both the targeted brain region and distant cortical and subcortical regions (e.g., Bohning et al., 1999; Bestmann et al., 2005). Though the distinction between local or remote effects of TMS may be inconsequential in some settings, in the case of Harris et al.'s (2002) findings, interpreting the effects of TMS as related to SI inhibition or PFC signal-to-noise modulation produce fundamentally different insights for neurocognitive models of STM storage. In such a case, it might be useful to, wherever possible, choose multiple stimulation sites (e.g., SI and PFC) and timings (e.g., early vs. late in the delay period at both sites) in order to design experiments that use TMS to conclusively elucidate the where and when of a given cognitive task in the brain.

It is likely that relatively limited activity in SI can produce effects downstream, given the feedforward nature of output connections from SI (Romo and Salinas, 2003). When discussing Harris et al.'s (2002) results, Romo and Salinas (2003) suggested that TMS was likely to produce localized effects in cortex for approximately 200 ms after application. This may be a conservative estimate; others have reported that the initial phase of increased neural activity can persist for approximately 500 ms after TMS application (Moliadze et al., 2003; Silvanto and Muggleton, 2008). However, given that the effects of activity in SII produced by vibrotactile stimuli can persist for several hundred milliseconds after stimulus offset (Romo and Salinas, 2003), as well as possible feedback loops within the vibrotactile memory system (e.g., Auksztulewicz et al., 2012), it appears plausible that effects of a TMS pulse in SI could produce longer-lasting effects downstream, and could produce substantial interference with VWM storage. Experimental tests of this hypothesis (possibly involving a combined TMS/ERP paradigm) should prove fruitful.

In the present study, we have suggested a way to integrate the TMS results of Harris et al. (2002) with the EEG and single-cell literatures. The present study also poses a challenge to the emergent-property model of working memory, and suggests a manner in which that model may be extended. Finally, and perhaps most intriguingly, the results of the current study are consistent with growing evidence that short-term and working memory may rely on different neural storage substrates, based on the salient property of the stimulus that is being maintained in memory; for example, in the present study, we have shown that simple tactile stimuli are stored in PFC (as are simple stimuli in other sensory modalities, see Bancroft et al., 2014) but complex tactile stimuli may be stored in sensory cortex (e.g., Zhou and Fuster, 1996).

### Conflict of interest statement

The authors declare that the research was conducted in the absence of any commercial or financial relationships that could be construed as a potential conflict of interest.
